# Downscaling NLDAS-2 daily maximum air temperatures using MODIS land surface temperatures

**DOI:** 10.1371/journal.pone.0227480

**Published:** 2020-01-16

**Authors:** William L. Crosson, Mohammad Z. Al-Hamdan, Tabassum Z. Insaf

**Affiliations:** 1 Universities Space Research Association, NASA Marshall Space Flight Center, Huntsville, AL, United States of America; 2 New York State Department of Health & University at Albany- State University of New York, Albany, NY, United States of America; George Mason University, UNITED STATES

## Abstract

We have developed and applied a relatively simple disaggregation scheme that uses spatial patterns of Land Surface Temperature (LST) from MODIS warm-season composites to improve the spatial characterization of daily maximum and minimum air temperatures. This down-scaling model produces qualitatively reasonable 1 km daily maximum and minimum air temperature estimates that reflect urban and coastal features. In a 5-city validation, the model was shown to provide improved daily maximum air temperature estimates in the three coastal cities, compared to 12 km NLDAS-2 (North American Land Data Assimilation System). Down-scaled maximum temperature estimates for the other two (non-coastal) cities were marginally worse than the original NLDAS-2 temperatures. For daily minimum temperatures, the scheme produces spatial fields that qualitatively capture geographic features, but quantitative validation shows the down-scaling model performance to be very similar to the original NLDAS-2 minimum temperatures. Thus, we limit the discussion in this paper to daily maximum temperatures. Overall, errors in the down-scaled maximum air temperatures are comparable to errors in down-scaled LST obtained in previous studies. The advantage of this approach is that it produces estimates of daily maximum air temperatures, which is more relevant than LST in applications such as public health. The resulting 1 km daily maximum air temperatures have great potential utility for applications such as public health, energy demand, and surface energy balance analyses. The method may not perform as well in conditions of strong temperature advection. Application of the model also may be problematic in areas having extreme changes in elevation.

## Introduction

Many methods have been developed over the past 30 years to ‘down-scale’ or ‘disaggregate’ remote sensing observations; these have been applied in diverse ways to many different physical variables, all with the intent of creating a higher-resolution version of a remotely-sensed variable. Excellent reviews of many of these methods are provided in [1,2]. As part of a project funded by the NASA Applied Sciences Public Health Program, which focuses on Earth science applications of remote sensing data for enhancing environmental public health decision-making, we have developed and applied a method for down-scaling gridded daily maximum air temperatures using spatial patterns of remotely-sensed LST. Heat-related death is currently the number one weather-related killer in the United States [3, 4]. Morbidity and mortality from heat events is expected to increase as a function of climate change [5, 6]. In order to improve the assessment of intra-urban variations in extreme heat risk, we developed and evaluated spatial statistical techniques for down-scaling 12 km meteorological re-analysis daily maximum air temperatures to a 1 km grid using MODIS LST data.

With the availability of improved high-resolution temperature data, more efficient mitigation strategies can be developed, improvements made in resource allocation, and morbidity and mortality from extreme heat events can possibly be reduced. High-resolution LST or air temperature products can enable more targeted assessment of areas and populations that are most likely to be affected by an extreme heat event [7–9]. The applications of these methods have the potential to be extended beyond public health and emergency response applications to many other environmental applications. For example, higher resolution assessment of intra-urban variations can enable stronger relationships to be established between urban surface heating and land cover/land use (LCLU) [10, 11]. Improved surface temperature and LCLU linkages can also provide information that can be used to improve inputs to watershed and land surface models [12, 13]. It has also the potential to be applied to management of urban forests and other ecosystems. More detailed information on surface heating variation among vegetation types can benefit urban tree planting strategies designed to cool public space and neighborhoods [8, 14, 15]. Hotspots could be identified within ecosystems that could be addressed with cooling or watering strategies that would enhance the long-term health of vegetated urban areas and provide useful information for efficient crop irrigation strategies. This down-scaling technique can also be used to enhance analyses of LCLU and associated changes in the surface energy budget in conjunction with climate projection scenarios [16].

Thermal data have been the focus of many down-scaling methods, due to the generally coarse spatial resolution of thermal remote sensing data relative to visible and near-infrared imagery. One of the first methods was described in [17], using Landsat Thematic Mapper (TM) Normalized Difference Vegetation Index (NDVI) 30 m data to down-scale TM 120 m thermal data over agricultural fields, based on the assumption of a monotonic relationship between NDVI and Land Surface Temperatures (LSTs). The concept was developed further by [18], who introduced the DisTrad method for disaggregating LST and applying it to aircraft data from the 1997 Southern Great Plains Experiment. This method calculates a regression model between coarse-scale LST and a predictor variable, which is applied to the high-resolution predictor data, thereby creating a high-resolution LST image. [18] used the DisTrad method to down-scale LST from 96 m to 24 m, using the inverse relationship between NDVI and LST. Validation of DisTrad using LST observations indicated that the model could down-scale LST data at the MODerate-resolution Imaging Spectroradiometer (MODIS) NDVI pixel resolution within ~1.5 K uncertainty. However, at the finer spatial scale of the Advanced Space-borne Thermal Emission Reflection Radiometer (ASTER)/Landsat, sub-pixel estimates did not show any skill, relative to an assumed uniform LST field.

Approaches similar to that applied in DisTrad have been used frequently in the past decade. A descendent of DisTrad called TsHARP was applied in [19] with NDVI input for an application in which thermal images at low resolution (180, 270, 450, 630, 810 and 990 m) were synthesized by aggregating 90 m ASTER data for agricultural fields. They then applied TsHARP to re-construct the original 90 m images using localized model fitting. Results using 180 m and 270 m inputs were quite positive, with Root Mean Squared Error (RMSE) values of 1.7–2.0 K, but results were not satisfactory when input values were at coarser resolutions. DisTrad was also used in [20] to disaggregate simulated LST data at 960 m, created by aggregating 30 m Landsat 7 ETM+ data, to resolutions ranging from 60 to 960 m using a regression approach based on a scale-independent linear relationship between LST and high-resolution impervious surface percentage. The RMSEs ranged from 1.1–3.8 K with the higher errors for finer resolutions.

Land use/land cover (LULC) data have been used in several approaches for down-scaling thermal data. For example, [21] applied a physical and a statistical model to disaggregate simulated 990 m ASTER thermal IR radiance observations down to 90 m, and validated the results vis-à-vis the original 90 m observations. The physical model that was applied was based on the functional relationship between thermal radiance and land cover fractions, while the statistical model estimated high-resolution thermal radiances directly based on ancillary high-resolution inputs. Model performance for the statistical and physical models was similar.

A new method was developed in [22] for down-scaling thermal radiance, addressing limitations of an earlier physical down-scaling method [21]. The newer method was found to greatly reduce spatial artifacts found in the original method, and also to improve down-scaling accuracy.

Recently, multi-spectral or multi-variable input images have been used as the basis for spatial down-scaling of thermal observations. A moving window analysis and a multiple regression model were used by [23] to enhance the resolution of the geostationary SEVIRI (Spinning Enhanced Visible and Infrared Imager) LST from approximately 4 km to 1 km, resulting in an average RMSE of 2.5 K. Independent variables in this model were the principal components of land cover, slope, aspect, sky-view factor, and Enhanced Vegetation Index, albedo, emissivity, the latter three being from MODIS 8-day composites. [24, 25] also down-scaled SEVIRI LST images down to 1 km using regression models, a neural network and regression trees. Inputs to the models included land cover, MODIS emissivity, elevation, and vegetation indices. The models were developed and applied globally (over the entire scene), which helps prevent errors near large water bodies. [26] statistically disaggregated geostationary LST data from resolutions of 3300–6700 m down to 100 m using several high-resolution predictors. Landsat TM and ETM+ thermal data were found to be the most valuable predictors, with an RMSE of 2.2 K. [27] down-scaled 1 km MODIS LST to a 250m resolution using a random forest regression model based on LST’s relationships with topographic variables, land cover data, and VIS/NIR reflectances, all of which are available at ~250m spatial resolution. Application of this model resulted in RMSE values of 1.4–1.9 K. In a similar study [28], MODIS LST was disaggregated to the Landsat spatial resolution using Landsat VIS/NIR data. Best results (average RMSE over four dates of 1.9 K between disaggregated and Landsat LST) were obtained by the method based on a linear regression between NDVI and LST.

These cited studies applied techniques to down-scale LST, and did not produce disaggregated air temperature products. One study that attempted to create a high-resolution air temperature dataset was [29], which used remotely-sensed LST to down-scale GDAS (Global Data Assimilation System) air temperatures. Also, [30] developed an algorithm to estimate 1 km near-surface air temperature using SEVIRI thermal data along with MODIS NDVI. In this model, the near-surface temperature lapse rate, parameterized in terms of albedo, downwelling shortwave flux, albedo and topography, was used to translate LST into air temperature. Application of this approach in multiple areas in Europe resulted in RMS deviations of ~ 2 K during the daytime.

Other efforts have focused on creating high-resolution meteorological datasets via spatial interpolation of surface observations, in which ancillary geographic data are used to create realistic spatial fields at fine spatial scales. Two such datasets are DayMet [31] and PRISM (Parameter-elevation Relationships on Independent Slopes Model) [32]. From the station data, DayMet and PRISM interpolate to a 1 km or 800m grid through sophistical spatial algorithms, which adjust temperatures based on surface elevation in the case of DayMet, and surface elevation and other physiographic factors such as coastal proximity, topographic facet orientation and vertical atmospheric layer in the case of PRISM. However, neither DayMet nor PRISM uses thermal remote sensing as an input.

## Materials and methods

### Datasets used

We calculated daily maximum air temperatures from hourly temperatures provided by the land-surface forcing fields for the North American Land Data Assimilation System Phase 2 (NLDAS-2), which have been derived from the analysis fields of the National Centers for Environmental Prediction (NCEP) North American Regional Reanalysis (NARR). NARR analysis fields are 32-km spatial resolution and 3-hourly temporal frequency. Those NARR fields are spatially interpolated to the finer resolution of the NLDAS-2 1/8 degree grid (~ 12–14 km) and then temporally disaggregated to create the NLDAS-2 hourly data. The details of the spatial interpolation, temporal disaggregation, and vertical adjustment of near-surface air temperature and specific humidity are those employed in NLDAS-1 as presented by [33]. The native NLDAS-2 hourly data used in this study were acquired as part of the mission of NASA’s Earth Science Division and archived and distributed by the Goddard Earth Sciences (GES) Data and Information Services Center (DISC) at https://disc.gsfc.nasa.gov/, as described in detail in [34].

Because NLDAS-2 air temperature data are based on 3-hourly NARR temperatures, daily maximum and minimum temperatures computed from NLDAS-2 will not exactly match observed values. In theory, NLDAS-2 maximum temperatures should be slightly lower, and minimum temperatures slightly higher, than temperatures based on continuous station observations, since extreme values between the 3-hourly NARR values will not be captured in the NLDAS-2 daily extrema. The NLDAS-2 forcing data are available from 1979 to the present. In this paper, we focus on results obtained using the down-scaling algorithm over the Conterminous United States (CONUS) for the warm season (May-September) of the years 2009–2011.

Although the resolution of NLDAS-2 is nominally 1/8 degree, it is in reality coarser because the NLDAS-2 variables are created via spatial interpolation of the 32 km NARR. Thus, most urban-scale features, such as the urban heat island, are not captured by NLDAS-2. In many applications, such as human health or electrical energy demand, finer-resolution data are required.

The MODIS data collections are derived from the NASA Aqua MODIS instrument, temporally spanning from 2002 until present. Aqua descends (ascends) the equator at 1:30 AM (1:30 PM) local time. The Aqua MYD11A1 product used in this study is the daily daytime LST product collected at spatial resolutions of 1 km over global land surfaces under clear-sky conditions (Level 3, Collection 5). This product is gridded in the Sinusoidal projection and is generated using the split-window algorithm [35], which uses bands 31 and 32 of MODIS's 36 spectral bands. As will be described in the next section, to compute seasonal means of LST, we used the MODIS/Aqua MYD11A2 product (an 8-day average composite of the aforementioned daily MYD11A1 product), which we obtained from the NASA Earth Science Data online system at https://earthdata.nasa.gov/. MYD11A2 is generated as the mean of all clear-sky LST values within the 8-day period, in an effort to minimize small-scale meteorological anomalies and missing data associated with the presence of clouds. We did not filter the MYD11A2 based on Quality Control flags.

Observations from five first-order National Weather Service (NWS) stations were used for validating results of the down-scaling algorithm. These data were obtained from the NWS Climate Services web site (https://w2.weather.gov/climate/index.php).

To create a higher-resolution version of NLDAS-2 air temperature data, we have implemented an algorithm that imposes 1 km spatial variations in MODIS LST onto the coarser-resolution NLDAS-2 air temperatures. The approach assumes that, in the absence of strong temperature advection, air temperature is driven by sensible heat flux from the surface, thus the spatial patterns of air temperature mimic the patterns of LST, although air temperature variations are much smaller in magnitude than corresponding LST variations. To account for this, our method applies normalized spatial departures of MODIS LST to disaggregate daily maximum NLDAS-2 air temperatures. Another assumption is that daily maximum air temperatures occur during the early- to mid-afternoon, near the time of the PM Aqua overpasses (1:30 PM local standard time). Both of these assumptions are generally appropriate for quiescent conditions associated with weak synoptic flow and no frontal passages, typical of the warm season at mid-latitudes.

### Description of algorithm

In this algorithm, the down-scaled daily maximum air temperatures for each day, T_DIS_, are calculated according to:

$$T_{DIS}=T_{L}+Z∙\sigma_{L},$$ (1)

where T_L_ and σ_L_ are the mean and standard deviation, respectively, of low-resolution (NLDAS-2) daily maximum air temperatures over a spatial neighborhood, and Z represents standardized MODIS LST departures, expressed as:

$$Z=(T_{H}-T_{H,mean}{)/\sigma }_{H},$$ (2)

where T_H_ = high-resolution (MODIS) LST and T_H,mean_ and σ_H_ are the mean and standard deviation, respectively, of MODIS LST over the neighborhood. Use of the standardized LST departure Z imposes the fine-scale (1 km) spatial distribution of temperature onto the coarser-scale NLDAS-2 maximum temperatures, i.e. Z is the down-scaling factor. Combining (1) and (2) gives:

$$T_{DIS}=T_{L}+(T_{H}-T_{H,mean}{)∙(\sigma_{L}/\sigma }_{H}).$$ (3)

In applying (3), it is first necessary to set the size of the spatial neighborhood. By calculating σ_L_ across the study area for different synoptic weather patterns, we determined that this variability can be very large due to local climatological gradients, particularly along the Pacific Coast, resulting in unrealistic down-scaled temperatures. Furthermore, using a neighborhood larger than one 12 km NLDAS-2 grid cell does not conserve the NLDAS-2 daily maximum temperatures. Therefore, we simplified the approach by parameterizing the ratio σ_L_/σ_H_ as a constant, R, in which case (3) reduces to:

$$T_{DIS}=T_{L}+R∙(T_{H}-T_{H,mean}).$$ (4)

The value of R was set based on evaluation of the variability of NLDAS-2 temperatures and MODIS LST over the CONUS. Evaluating this variability in different CONUS regions, values of R were found to be high in coastal and mountainous areas, but in other regions, representing the great majority of the study area, values are generally less than 1.0. Based on validation against surface temperature observations, we set R to 0.50 for maximum temperature, realizing that this solution is not ideal for all locations. That R < 1 reflects the fact that variations in 2 m air temperature are generally less than variations in LST. In (4), T_L_ is defined as the 12 km NLDAS-2 daily maximum air temperature, i.e. there is no further averaging to a neighborhood.

The term (T_H_−T_H,mean_) represents the geographic temperature pattern, i.e. the local (1 km) LST departures from the mean LST over an NLDAS-2 12 km grid cell. Ideally, this would reflect both quasi-permanent temperature patterns, which are due primarily to land use, as well as variability operating at shorter time scales, such as non-uniform rainfall patterns and vegetation phenology. The MODIS LST 8-day composite (Aqua product MYD11A2) is in theory well-suited to provide this information. In practice, though, we found that missing LST observations due to cloud cover make it impractical to calculate (T_H_−T_H,mean_) at an 8-day time scale. In fact, we found it necessary to average LST observations over periods of several months to provide a robust estimate of (T_H_−T_H,mean_). In order to avoid the problems of missing data, we calculated this term from all 8-day daytime (1:30 PM local standard time) composites from the warm season (May-September) as the mean of the upper half of non-zero values at each grid point. The upper half of the distribution is used to eliminate missing data as well as occasional erroneously low MODIS temperatures that occur due to partial cloud contamination. The resulting mean (T_H_−T_H,mean_) was applied for all down-scaling analysis for the respective year.

The down-scaled maximum air temperatures are computed for each day in May-September using (4). In this calculation, T_L_ is given by the NLDAS-2 daily maximum air temperature, R is constant (0.5) and (T_H_−T_H,mean_) is an annual mean value. The approach is that the relative spatial pattern (i.e. 1 km anomalies with respect to a neighborhood mean) is fixed for the year, but the daily variations in air temperature are represented by the NLDAS-2 maximum temperatures.

**Fig 1:**
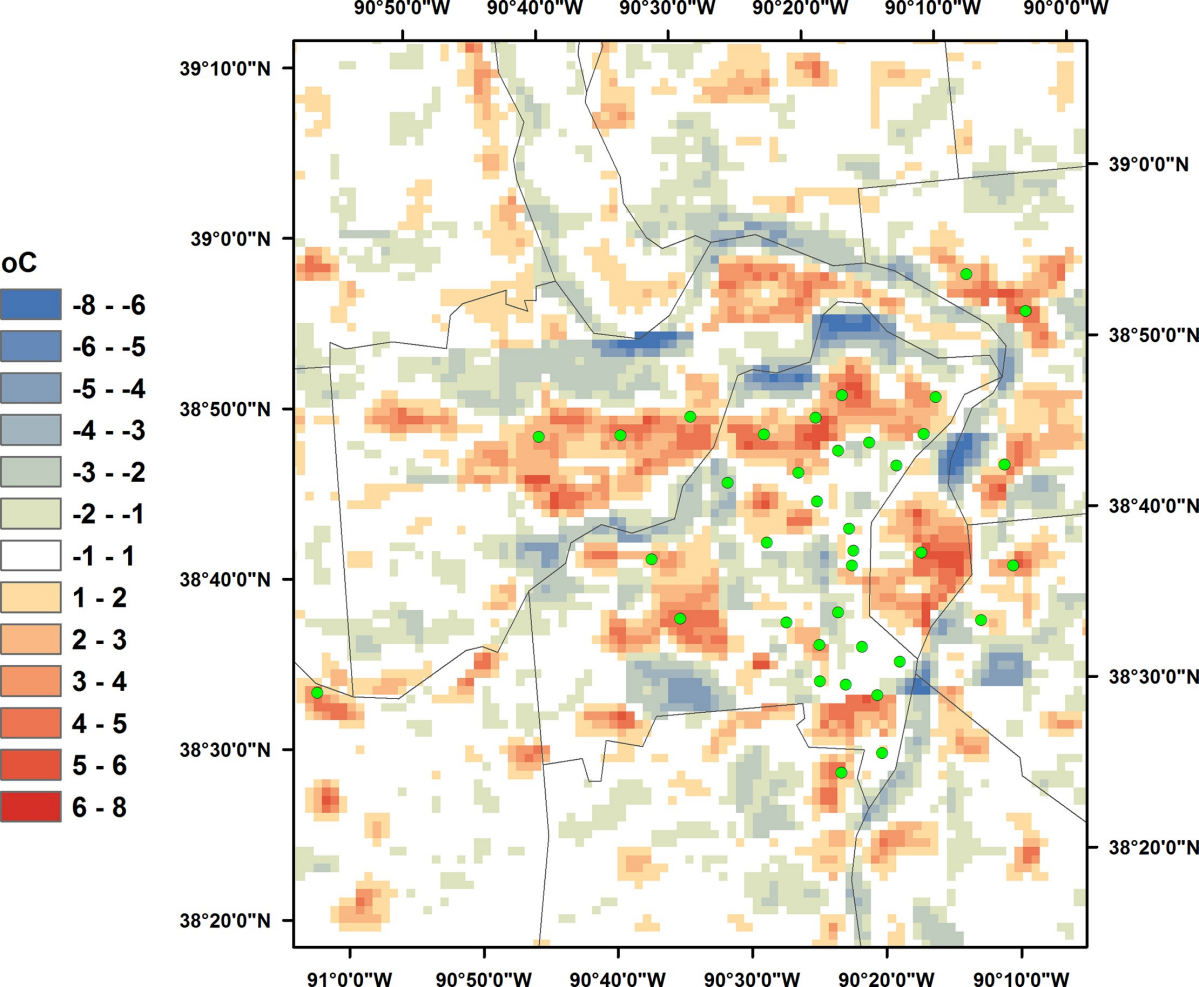
MODIS LST departures for the St. Louis, MO area for May-September 2009 for daily maximum temperatures. City centers are indicated by green circles.

Examples of MODIS LST maximum temperature departures (T_H_−T_H,mean_) are shown in Fig 1 for the St. Louis, MO area. Cool areas are characterized by negative (blue) values, while warmer areas exhibit positive (red) values. Values between -1 and +1 C^o^ are shown in white. During the daytime, urban areas are warmer than the surroundings, and the river is cooler.

The temperature departures shown in Fig 1 clearly capture urban-rural temperature variations, which are not evident in composites of 12 km NLDAS-2 air temperatures. This attribute of the summer MODIS LST composite makes it well-suited for use in a down-scaling method, particularly where the intended purpose is to develop daily heat metrics that represent these spatial patterns of heat. Use of this annual LST product in the down-scaling algorithm requires the assumption that relative spatial patterns of air temperature at the sub-NLDAS-2 scale are relatively constant from day to day within the respective season. This temperature departure array, multiplied by R (Eq 4) provides the adjustments, at each 1 km grid cell, to the 12 km NLDAS-2 daily maximum air temperature.

## Results

### Examples of down-scaled daily maximum temperatures for urban areas

**Fig 2:**
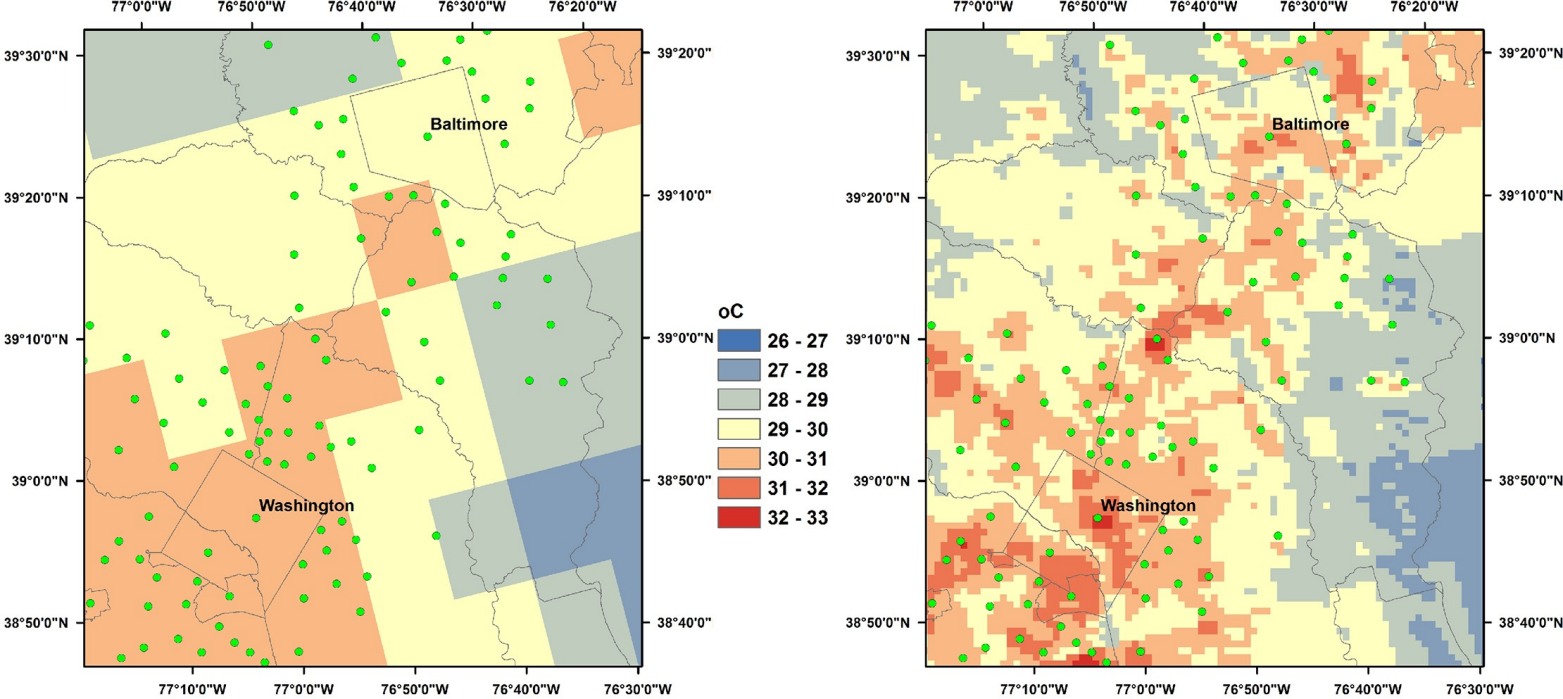
Daily maximum temperature estimates for the Washington-Baltimore region, 12 July 2009. Left: 12 km NLDAS-2. Right: 1 km down-scaled estimates. City centers are indicated by green circles.

**Fig 3:**
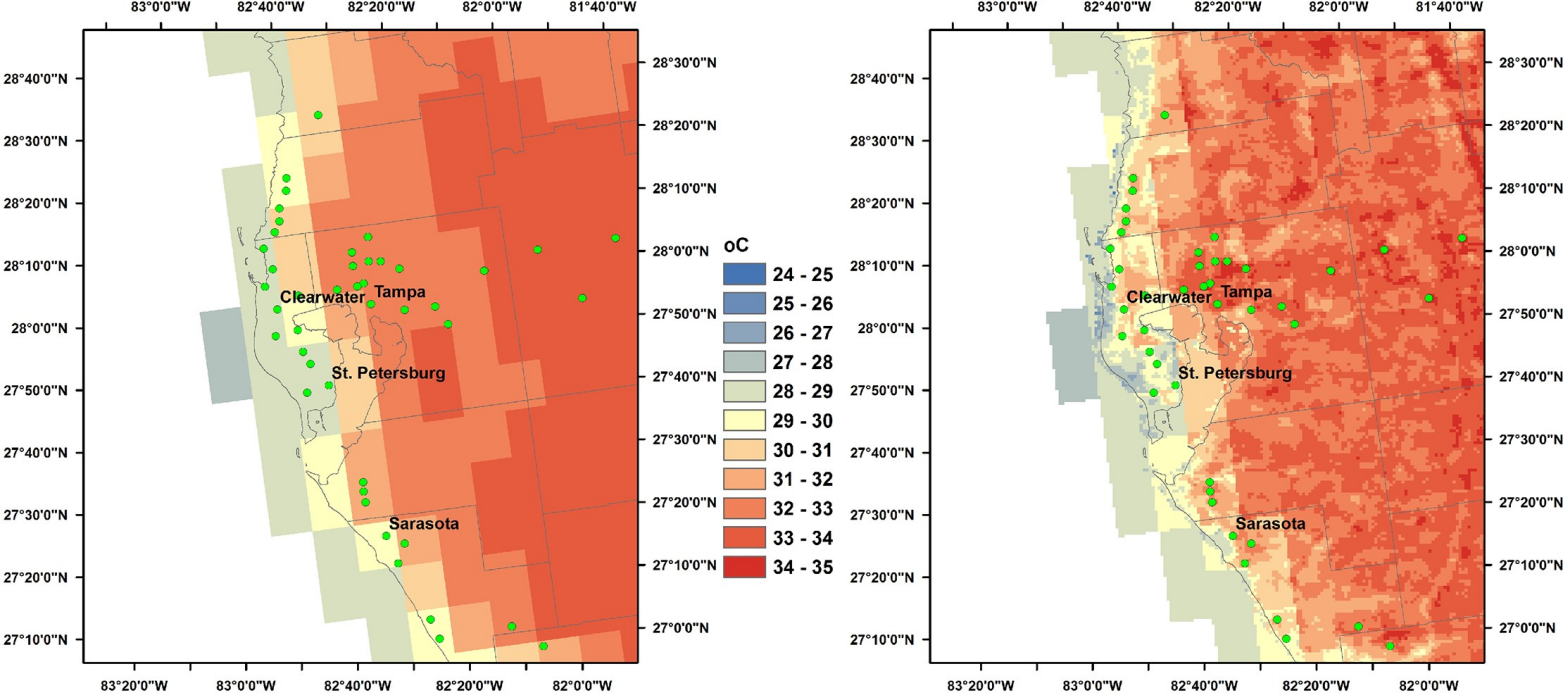
Daily maximum temperature estimates for the Tampa Bay, Florida region, 12 July 2009. Left: 12 km NLDAS-2. Right: 1 km down-scaled estimates. City centers are indicated by green circles.

Application of Eq (4) using NLDAS-2 daily temperatures, along with MODIS temperature departures calculated for each annual warm season, produces estimates of down-scaled daily maximum temperatures over the CONUS. Figs 2 and 3 show examples of NLDAS-2 and down-scaled maximum temperatures for two regions for 12 July 2009. These temperature maps are shown to illustrate, for a single day, the spatial patterns of the down-scaled temperatures vis-à-vis the lower resolution NLDAS-2 temperatures, but are separate from the validation analysis presented in the following section. Fig 2 compares 12 km NLDAS-2 with 1 km down-scaled maximum temperatures for the Washington-Baltimore region. In the NLDAS-2 image (left panel), a weak warm corridor extends from Washington toward Baltimore, but the resolution is insufficient to capture urban-rural temperature differences. In the down-scaled temperature field (right panel), much more detail is evident, with warmer areas being well-correlated with city centers, shown in green circles.

Fig 3 shows the results of the down-scaling algorithm for the Tampa Bay, Florida area. For daily maximum temperature, the original NLDAS-2 12 km values show a general gradient from the coast to inland areas, while the down-scaled version shows much more variability. Notably, the string of cities north of Clearwater are distinctly warmer than their surroundings, and the city of Tampa and suburbs are also a prominent warm region.

### Validation

Validation of the algorithm was performed for five cities, chosen to represent a range of climate conditions. Three of these (San Francisco, Washington and Baltimore) are near coastlines, areas where we hypothesize that the down-scaling algorithm will improve the agreement with observed maximum daily temperatures. The other two cities (St. Louis and Atlanta) are inland, although St. Louis lies on a major river. We performed validation by statistically comparing daily maximum temperatures with observations from the first-order National Weather Service stations in these cities. We first extracted the NLDAS-2 12 km and down-scaled (1 km) estimates for the locations of the observation stations from the respective grids and computed mean differences and Root Mean Square Differences (RMSD) over June-August of the years 2009–2011 (n = 276 for each city). Down-scaled estimates agree better than the original 12 km NLDAS-2 temperatures with temperature observations for three of the five sites (San Francisco, Washington and Baltimore), with the improvement being substantial for San Francisco (Table 1). For Atlanta and St. Louis, results are slightly worse for the down-scaled estimates, compared to the original NLDAS-2 temperatures. Overall, errors in the down-scaled maximum air temperatures are comparable to errors in down-scaled LST obtained in the previous studies cited in Section 1.

**Table 1:** Daily maximum temperature validation statistics for five cities, averaged over June-August of 2009–2011 (n = 276 for each city). Bold text indicates the superior performance for each city and each metric.

City	Mean Difference (deg. C)		RMSD (deg. C)	
	NLDAS-2	Down-scaled	NLDAS-2	Down-scaled
Atlanta, GA	**0.56**	1.09	**1.87**	2.05
San Francisco, CA	-3.69	**-1.79**	4.44	**3.05**
Washington, DC	-0.53	**-0.25**	1.52	**1.45**
Baltimore, MD	-1.11	**-0.17**	1.96	**1.63**
St. Louis, MO	**0.02**	1.45	**1.96**	2.38
5-city Mean	-0.95	**0.07**	2.35	**2.11**

The Pacific Coast poses a particularly difficult problem for maximum temperature estimation, due to the extreme gradients from the immediate coast to inland areas. In summer, daytime temperatures can vary by more than 20 ^o^C over distances of less than 50 km. Due to the large true temperature variability within an NLDAS-2 grid cell, the temperature at an observation site can differ substantially from the NLDAS-2 temperature, which represents a mean over a 12x12 km area. This is seen in the large errors in the NLDAS-2 maximum temperatures for San Francisco (Table 1). The down-scaling approach greatly reduces these errors. Along with positive results for coastal cities of Washington and Baltimore, this illustrates a high potential value for the down-scaling algorithm in coastal areas. Conversely, in inland, non-mountainous cities like St. Louis and Atlanta, spatial variability of air temperature is relatively low, and down-scaling air temperatures are not very different from the original 12 km NLDAS-2 temperatures. Thus, the down-scaling model is not likely to result in improved maximum air temperature estimates in these types of regions.

In a separate study, we conducted additional validation of the down-scaling model for New York City and Florida. Using neighborhood-scale data from the New York City Community Air Survey for 2009–2010, we found that the model greatly improved correlations with *in situ* summertime daily minimum temperatures [36]. Based on data from 85 weather stations in Florida, we found that down-scaling of daily maximum air temperatures reduced the mean differences between estimates and observations by more than 30%, compared to the 12 km NLDAS-2 maximum temperatures (unpublished study).

## Discussion

We have developed and applied a relatively simple disaggregation scheme that uses spatial patterns of Land Surface Temperature from MODIS warm-season composites to improve the spatial characterization of daily maximum air temperatures. The relationship between LST and air temperature is complex, being affected by land cover, boundary layer stability, and synoptic weather conditions [37]. Accordingly, the method relies on a few assumptions about the relationship between LST and air temperature, which are generally met under tranquil synoptic conditions during the warm season. This down-scaling model produces qualitatively reasonable 1 km daily maximum air temperature estimates that reflect urban and coastal features. In a limited (5-city) validation, the model was shown to provide improved daily maximum air temperature estimates in three cities, with marginally less accurate estimates in the other two cities, compared to 12 km NLDAS-2 temperatures.

The approach has some limitations but appears to have great utility for estimating air temperatures at a fine spatial scale, with particular value in daily maximum temperatures in urban and coastal areas. The resulting daily maximum air temperatures, at a 1 km spatial resolution, have great potential utility for applications such as public health, energy demand, and surface energy balance analyses. The method may not perform as well in conditions of strong temperature advection. Application of the model also may be problematic in areas having extreme changes in elevation within a 12 km NLDAS-2 grid cell, even though temperature variations at the 1 km scale are captured by use of MODIS LST. This problem is likely due to the fact that the assumed global value of the ratio R is not appropriate in the presence of large elevation difference. However, temperature information at lower resolutions, i.e. 12 km NLDAS-2 data, are also inaccurate in such areas, and in fact it is very likely that the down-scaled maximum temperature estimates are an improvement over the NLDAS-2 estimates, despite the simplified approach of applying a constant R.

Additional rigorous model validation is needed to determine how well the algorithm performs across a range of synoptic conditions, across different geographic regions, and in complex topography. This will require validation to be performed for many more cities than the five selected for analysis and discussed herein.

Heat Index (HI), wind chill, and other temperature-related metrics can be calculated at the 1 km scale, with some assumptions. For example, the daily maximum HI can be calculated from the down-scaled daily maximum temperature, along with the relative humidity at the time of the maximum temperature (one of the available NLDAS-2 12 km products), assuming that the maximum HI occurs simultaneously with maximum temperature, a reasonable but not perfect assumption. Nighttime and daytime wind chills can be computed from the down-scaled maximum and minimum temperatures and coincident wind speeds.

One potential improvement to this model is to derive and apply a spatially-varying R ratio; this would allow the model to capture the higher variability of temperatures in mountainous and coastal areas. Toward this end, the model could be modified to use a topographic variable as an input. Other geospatial inputs such as land cover, which is a quasi-static surface property, or more dynamic properties such as soil moisture could also be used as inputs. However, there is a risk of an over-fitted model due to the strong cross-correlations between some of these variables.

A natural follow-on to this research would be to evaluate potential methods to improve spatial delineation of risk from extreme heat events in urban environments by integrating socio-demographic risk factors with LST estimates derived from thermal remote sensing data.

## Conclusions

The methods described herein have great potential for public health research, namely by increasing our ability to determine exposure to excessive temperatures at close to a ‘neighborhood’ scale. A potential use of these methods and data is to augment current Heat Watch/Warning Systems (HWWS) with NASA remotely-sensed data and models used in conjunction with socioeconomic and heat-related mortality data. The current HWWS do not consider intra-urban spatial variations in risk assessment, but this variability can be estimated using the approach developed here. The methods described here can be modified for such purposes by merging gridded meteorological forecasts with remotely-sensed LST observations, such as 1 km MODIS LST used in this analysis, or finer-scale thermal observations from Landsat or similar sensors. The algorithm can be applied in near real-time, limited by the ~3-day latency of NLDAS-2 availability.

This method for producing high-resolution daily maximum and minimum air temperatures can be applied on a regional to national scale, with some exceptions as noted above. Additional verification of the algorithm is needed, focusing on mountainous and coastal regions, in order to improve estimates in these areas. An executable for running the down-scaling model developed by the authors is available from the New York State Environmental Public Health tracking program by contacting Tabassum.insaf@health.ny.gov or epht@health.ny.gov.
